# 966 CLABSI Prevention in Focus: Shaping a Future of Zero

**DOI:** 10.1093/jbcr/iraf019.497

**Published:** 2025-04-01

**Authors:** Catrina Cullen, Anna Ehoff, Katrina Whiting, Nicole Kopari, Crystal Arias

**Affiliations:** University of California San Francisco, Fresno, Leon S. Peters Burn Center; University of California San Francisco, Fresno, Leon S. Peters Burn Center; University of California San Francisco, Fresno, Leon S. Peters Burn Center; University of California San Francisco, Fresno, Leon S. Peters Burn Center; University of California San Francisco, Fresno, Leon S. Peters Burn Center

## Abstract

**Introduction:**

Central-line-associated bloodstream infections (CLABSIs) are found to cause longer hospital stays, increased healthcare costs, and higher morbidity rates for patients. Burn patients are especially susceptible to CLABSIs because they often require central lines for an extended amount of time. Also, the central lines may need to be placed on or near the wounds, which limits our ability to maintain an occlusive dressing. These factors place burn patients at a high risk for CLABSIs. Our goal is to find a way to decrease or eliminate the occurrence of CLABSIs in burn patients.

**Methods:**

Protocols were initiated that required bedside staff to perform a CHG bath on non-burned areas of skin on a daily basis for all patients with a central line. Burn patients who are hemodynamically stable must have a linen change at least once per shift to limit exposure to infectious agents. Monthly monitoring and chart audits are performed to ensure compliance with protocols and offer opportunities for further education for staff when necessary.

**Results:**

CLABSI rates have gone from 3 in 2022 and continued to zero as of August 2024.

**Conclusions:**

Daily CHG baths and twice-daily linen changes have decreased exposure to infectious agents in burn patients, thus limiting the number of CLABSIs present. Auditing documentation allows us to ensure protocol compliance and assess the need for further education. Furthermore, no longer rotating insertion sites for central lines have decreased the likelihood of introducing new microbes into the bloodstream during insertion.

**Applicability of Research to Practice:**

Limiting the number of CLABSIs will help reduce patients’ hospital stays, leading to lower mortality rates and better patient outcomes overall.

**Funding for the Study:**

N/A

